# Impact of Fast High-Intensity versus Conventional Light-Curing Protocol on Selected Properties of Dental Composites

**DOI:** 10.3390/ma14061381

**Published:** 2021-03-12

**Authors:** Sufyan Garoushi, Lippo Lassila, Pekka K. Vallittu

**Affiliations:** 1 Department of Biomaterials Science, Turku Clinical Biomaterial Center—TCBC, Institute of Dentistry, University of Turku, 20500 Turku, Finland; liplas@utu.fi (L.L.); pekval@utu.fi (P.K.V.); 2 Welfare Division, Oral Health Care, 20101 Turku, Finland

**Keywords:** 3s PowerCure, curing protocol, Tetric PowerFill, physical properties, composite resin

## Abstract

To study the influence of fast high-intensity (3-s) and conventional (20-s) light curing protocols on certain physical properties including light-transmission and surface wear of two nano-hybrid composite resins (Tetric PowerFill and Essentia U) specifically designed for both curing protocols. According to ISO standards, the following properties were investigated: flexural properties, fracture toughness and water sorption/solubility. FTIR-spectrometry was used to calculate the double bond conversion (DC%). A wear test using a chewing simulator was performed with 15,000 chewing cycles. A tensilometer was used to measure the shrinkage stress. Light transmission through various thicknesses (1, 2, 3 and 4 mm) of composite resins was quantified. The Vickers indenter was utilized for evaluating surface microhardness (VH) at the top and the bottom sides. Scanning electron microscopy was utilized to investigate the microstructure of each composite resin. The light curing protocol did not show a significant (*p* > 0.05) effect on the mechanical properties of tested composite resins and differences were material-dependent. Shrinkage stress, DC% and VH of both composite resins significantly increased with the conventional 20 s light curing protocol (*p* < 0.05). Light curing conventional composite resin with the fast high-intensity (3-s) curing protocol resulted in inferior results for some important material properties.

## 1. Introduction

As consequence of the recent development in their physical, bonding and esthetic properties, composite resins have become the most popular materials for direct restorations [1]. It was stated that regular dental practitioners working in public dental services consume over half of their working hours applying direct light cure restorations [2]. Accordingly, dental practitioners are always searching for an effective method to handle their clinical procedures involving quick-set materials and reduced practical steps to decrease total time and costs. This trend or wish of shortening the restorative procedure time leads to competition between dental manufacturers to develop faster restorative techniques with shorter practical steps and light curing time. Nevertheless, this requires dentists to improve their knowledge of the new materials/equipment and their safe practical applications [3,4,5].

Light-emitting diode (LED) curing lights nowadays include halogen, plasma arc, and laser lights in the dental market. The emission spectrum from broad-spectrum (400–500 nm) LED curing-lights (3rd generations) should have the capacity to stimulate all the photoinitiators commonly utilized in composite resins [6]. This generation of light curing units have also been defined as “multi-wave” or “multi-peak” dental light curing units. The in-situ photo polymerization of composite resins requires the use of a special system suitable for generating optimum polymerization through a significant depth of colored material, using suitable light curing times (≈20 s). Typically, for most of the commercially available composite resins, camphorquinone (CQ) as a type II photoinitiator, together with tertiary amine as co-initiator, is used [7]. Directly after photon absorption, the photoinitiator system begins a free-radical polymerization process. Previous investigations have shown that methacrylate-based composite resins are well-known to exhibit uncompleted cross-linking and cure with the majority of unconverted C=C double bonds staying inside the matrix, leaving around 10% of leachable unpolymerized monomers, causing compromised physical stability [8].

The use of radical polymerization to control the polymer network architecture was suggested as a strategy to enhance the physicochemical stability of the composite resins. Through replacing the growth of radical chain with a step-like polymerization, it is possible to obtain a more homogenous polymer network with enhanced properties. Through the inclusion of of the β-allyl sulfone reagent in the composite resins, a reversible addition-fragmentation chain transfer (AFCT) polymerization mechanism was able to be achieved [9]. Research has shown that the network architecture was improved during polymerization by integrating AFCT reagent into a dimethacrylate matrix, leading to enhanced network homogeneity and physical properties [9,10]. This kind of modification was recently used in a bulk-fill composite resin material in the context of a restorative system, named the 3 s PowerCure system (Tetric PowerFill, Ivoclar Vivadent) [10,11,12,13]. This composite resin was designed to be light cured in 3 s, with high-irradiance light emitted by a multi-wave light curing unit (Bluephase PowerCure, Ivoclar Vivadent).

Despite the fact that the polymerization reaction is ultimately chemical, a few important points of the reaction are still regulated by dentists. The curing time and radiant energy control the sum of energy received to activate the photoinitiators and consequently the quality and rate of curing [1,14]. Insufficient delivery of light curing power could create a low level of monomer conversion and real weakness, leading to more problems like bulk fracture, marginal deterioration and recurrent caries [15,16]. The influence of using this fast and high-intensity light curing protocol has still not been investigated completely. The question arises whether one may use this new 3 s PowerCure protocol (fast high-intensity light curing) with a conventional composite resin, or use a conventional light curing protocol (20 s) with the Tetric PowerFill composite resin. This possible clinical scenario might lead to undesirable clinical outcomes and thus laboratory investigations are vitally needed. This possibility exists because the dental practitioner can only examine the composite resin’s top surface. It is possible that a composite resin may seem rigid and well-polymerized, but the majority of the composite resin below the surface could be under-cured.

Accordingly, in this study, we aimed to investigate the impact of fast high-intensity (3 s) and conventional (20 s) light curing protocols on certain physical characteristics including light transmission and surface wear in two nano-hybrid composite resins (Tetric PowerFill and Essentia U), specifically designed for both curing protocols. The null hypotheses were that the curing protocol and material type would have no effect on the tested properties of the composite resin.

## 2. Materials and Methods

### 2.1. Composite Materials and Light Curing Protocols

Two packable nano-hybrid composite resins, one conventional (Essentia U) and one bulk-fill (Tetric PowerFill), were used in this study (Table 1). Tetric PowerFill is intended for a fast high-intensity light curing protocol (3 s) and Essentia is designed for conventional light curing protocol (20 s). A violet-blue LED curing unit (Bluephase PowerCure, Ivoclar Vivadent, Schaan, Liechtenstein; emission wavelength range: 400–500 nm; irradiance approximately 2400 mW/cm^2^) was used for the fast high-intensity light curing protocol (3 s). An Elipar LED curing unit (TM S10, 3M ESPE, Seefeld, Germany; emission wavelength range: 430–480 nm; irradiance approximately 1600 mW/cm^2^) was used for the conventional light curing protocol (20 s). The light irradiance values and wavelength spectra were measured using a Marc Resin Calibrator (BlueLight Analytics Inc., Halifax, Canada). Each composite resin was light cured by means of the two mentioned protocols.

### 2.2. Mechanical Tests

From each composite resin, 3-point bending sticks (2 × 2 × 25 mm^3^) were prepared in half-split molds (stainless steel) between transparent Mylar sheets. Half of the specimens were polymerized using the 3-s light curing protocol and the other half were polymerized using the 20-s light curing protocol. Polymerization of the composite resins was performed from both sides of the stainless-steel mold at five different positions. The specimens from each composite resin were either stored in water (for one month) or dry (for one day) at 37 °C before testing. In line with ISO 4049 [17] (test span: 20 mm, crosshead speed: 1 mm/min, indenter: 2 mm diameter), the 3-point bending test was assessed. All specimens (*n* = 6/group) were loaded into a universal testing machine (LRX model, Lloyd Instruments Ltd., Fareham, UK) and PC software (Nexygen 4.0, Lloyd Instruments Ltd.) to record the load-deflection curves. Flexural strength (ơ_f_) and flexural modulus (E_f_) were measured according to these equations (ISO 10477:1992) [18]:

ơ_f_ = 3F_m_L/(2bh^2^)     E_f_ = SL^3^/(4bh^3^) (1)

where L is the length of span (20 mm), F_m_ is the applied load (N) at the highest point of a load-deflection curve, h is the thickness of test specimens, and b is the width of the test specimens.

In line with the ASTM E1820-05 standard method [19], single-edge-notched-beam specimens (2.5 × 5 × 25 mm^3^) were made to determine the fracture toughness. A half-split mold of stainless steel was used, which allowed the removal of the specimen without force. A centrally manufactured slot was designed in the mold, extending up to its mid-height, allowing the central position of the notch and the optimization of the crack length (x) to be half the height of the specimen. In one increment, the composite resin was packed into the mold, placed over a Mylar-strip-covered glass slide. A sharp and centrally positioned crack was created prior to polymerization by inserting a straight edged steel blade into the prefabricated slot. With the two tested light curing protocols, polymerization of the composite resin was carried out in five different positions. Before polymerization, the upper side of the mold was covered with a Mylar strip and a glass slide from both sides of the blade. After removal from the mold, the opposite side of each specimen was also polymerized. Before testing, the specimens from each group (*n* = 6) were stored dry in an incubator (37 °C) for 24 h. The specimens were tested in a 3-point bending mode with a crosshead speed of 1.0 mm/min on a universal material testing machine. The fracture toughness (FT) was measured through the following formula:

(2) Kmax=⌈P·LB·W32⌉·f(x)

where

f(x)=32·x12·[1.99−x·(1−x)·(2.15−3.93·x+2.7·2)]/2·(1+2·x)·(1−x)32

Here, *L* is the length of span (2 cm), *B* is the thickness of the specimen in centimeters (cm), *P* is the maximum load in kilonewtons (kN), *W* is the width of the specimen (depth) in cm, *x* is a geometrical function dependent on a/W and *a* is the length of the crack in cm.

### 2.3. Double Bond Conversion

Fourier transform infrared spectroscopy (FT-IR) (Spectrum One, Perkin-Elmer, Beaconsfield Bucks, UK) with an attenuated total reflectance (ATR) accessory tracked the double bond conversion (DC%) during and after the photoinitiation of polymerization. Composite resins were analyzed in a mold that was 1.5 mm thick and 4.5 mm in diameter. The spectrum of the unpolymerized sample was first placed and measured in the mold. Using the two tested curing protocols, the specimen was then irradiated via an upper glass slide. Next, the specimen was irradiated via an upper glass slide using the two tested curing protocols. When irradiated, the specimen was scanned for its FT-IR spectrum (monitored for 15 min). The DC% was measured from the aliphatic C=C peak at 1636 cm^−1^ and normalized against the aromatic ring C=C peak at 1608 cm^−1^ based on the following equation:

(3) $$DC=\frac{{\left(A_{C=C}/A_{ph}\right)}_{0}-{\left(A_{C=C}/A_{ph}\right)}_{t}}{{\left(A_{C=C}/A_{ph}\right)}_{0}}\times 100\%$$

where *A_C=C_* and *A_ph_* are the absorbance peak area of methacrylate C=C at 1636 cm^1^ and the aromatic ring at 1608 cm^−1^, respectively; (*A_C=C_/A_ph_*)_0_ and (*A_C=C_/A_ph_*)*_t_* represent the normalized absorbency of the functional group at the radiation time of 0 and t, respectively; and DC is the conversion of methacrylate C=C at a given radiation time. Five trials were carried out for each group.

### 2.4. Shrinkage-Stress Measurement

Glass fiber-reinforced composite (FRC) rods with length of 4 cm (Ø 4 mm) were ground flat at their ends with 180 grit silicon carbide sandpaper. A universal testing machine was tightly attached to two FRC rods and composite resin was inserted between the surfaces of the FRC rods. The specimen height was set at 1.5 mm. The tip of the light guide of the light curing units (Bluephase and Elipar) was in close contact from both sides with the composite resin specimen. At room temperature (22 °C), contraction forces were monitored for 5 min. By dividing the shrinkage force by the cross-section area of the FRC rod, shrinkage stress was measured. At the end of the shrinkage stress/time curve, the maximum shrinkage stress (SS) value was extracted from the plateau. For each group, five specimens were evaluated.

### 2.5. Surface Microhardness

The surface microhardness of 2- and 4-mm thick specimens was assessed using a Struers Duramin hardness microscope (Struers, Copenhagen, Denmark) with a 40× objective lens and a load of 1.96 N, subjected for 10 s. On each specimen (*n* = 5/group), three measurements were recorded at the top side and three at the bottom side. The diagonal length impressions were calculated and Vickers values were converted into hardness values by the machine. Surface hardness was measured using the following formula:

(4) $$H=\frac{1854.4\times P}{d^{2}}$$

where H is Vickers hardness in kg/mm^2^, *d* is the diagonal length in μm and *P* is the load in grams. The ratio between VH at the bottom and top surfaces was measured.

### 2.6. Two-Body Wear

In an acrylic resin block, four specimens of each composite resin were prepared for localized wear testing. For each group, a long cavity (20 mm length × 10 mm width × 2 mm depth) was prepared and then composite resin was applied in a bulk layer into the prepared cavity and covered with Mylar strips and glass slides before being light cured according to the tested protocols in five different positions. Using a series of #1200-to #4000-grit silicon carbide sheets, the surfaces were then polished flat. The 2-body wear test was performed one day after storage in water (37 °C) using the chewing simulator (CS-4.2, SD Mechatronik, Feldkirchen-Westerham, Germany), which has two chambers simulating vertical and horizontal motions with water simultaneously. Every chamber has a lower plastic sample holder in which the composite resin specimen was embedded and an upper sample holder that can fasten the loading tip (antagonistic) with a screw. The regular loading tips (Steatite ball, Ø 6 mm) of the manufacturer were embedded in the upper sample holders in acrylic resins and were then fixed with a fastening screw. A weight of 2 kg was used, equivalent to 20 N of chewing force and 15,000 loading cycles, with a frequency of 1.5 Hz. On the surface of each specimen, the wear patterns (*n* = 6/group) were profiled with a 3D optical microscope (Bruker Nano GmbH, Berlin, Germany) using Vision64 software. Total wear depth values (μm) were measured from different points, reflecting the average of the lowest or deepest points of all profile scans.

### 2.7. Surface Roughness

The surface roughness of each composite resin was measured before and after the wear test using a 3D non-contact optical profilometer (Bruker Nano GmbH). A 5× objective lens and a 0.5 multiplier was used, with a back scan and length parameters of 20 µm and 60 µm in VSI/VXI mode to obtain a 3D rendering of the specimen surfaces (*n* = 6/group). Software (Vision 64) to generate surface areas and roughness parameters was used. The particular parameter of interest was considered to be the roughness average (Ra) measured from a mean line within the sampling length.

### 2.8. Light Transmission

For each composite resin, four various incremental thicknesses (1, 2, 3 and 4 mm) were viewed (*n* = 5/group). The specimens were made in cylindrical Teflon molds that were open at the bottom and the top sides and cured according to the tested light curing protocols (3 and 20 s) by handling the curing unit (Bluephase and Elipar) directly, perpendicular and centered on the specimen’s surface utilizing a mechanic arm. During the curing of the specimens, a spectrometer (MARC Resin Calibrator, Blue Light Analytics Inc., Halifax, Canada) calculated in real time the transmitted irradiance at the bottom of the specimens. Various thicknesses of the ring mold without composite resin were used as controls. The MARC system contained a NIST-reference miniature spectrometer (USB4000, Ocean Optics, Dunedin, FL, USA) with a 3648-element linear CCD array detector (TCD1304AP, Toshiba, Tokyo, Japan). The sensor was a CC3 cosine corrector (diameter 4 mm) designed to collect radiation (light) at around 180°, eliminating optical interface problems associated with the light collection sampling geometry. For data recording, irradiance at wavelengths of 360–540 nm was considered. A thin transparent plastic foil was used to protect the sensor of the spectrometer, as well as the tip of the light guide, and to get rid of the oxygen inhibition layer.

### 2.9. Water Sorption and Solubility

Water sorption and solubility were assessed using eight specimens from each group, each of them stored in 120 mL water for 30 days at 37 °C. The dry weight (md) of the specimens was weighted with a balance (Mettler A30, Mettler Instrument Co.,Highstone, NJ, USA), with an accuracy of 0.1 mg. During the water immersion, the weights (mw) of the specimens were assessed at 1, 2, 3, 7, 14, 21 and 30 days. Solubility weight (mh) was assessed after the dehydration was stabilized at 60 °C in air. Water sorption and solubility were measured as follows:

Water sorption % = (mw − mh)/md × 100%

Water solubility% = (md − mh)/md × 100%

### 2.10. Microscopic Analysis

Scanning electron microscopy (SEM, LEO, Oberkochen, Germany) afforded the characterization of the microstructure of the material surface of the investigated composite resins (magnifications: 1000× and 2500×). Polished specimens (*n* = 2) from each composite were stored in a desiccator for one day. Then, they were coated with a gold layer using a sputter coater in a vacuum evaporator (BAL-TEC SCD 050 Sputter Coater, Balzers, Liechtenstein) before the SEM examination. SEM analysis was conducted at an operating voltage of 8 kV and a working distance of 13 mm.

### 2.11. Statistical Analysis

Data were statistically examined with a 2-way analysis of variance (ANOVA) followed by Tukey’s post hoc analysis test (α = 0.05) to determine the differences between the groups using SPSS version 23 (SPSS, IBM Corp., Armonk, NY, USA). Curing protocol and material type were independent variables.

## 3. Results

**Figure 1:**
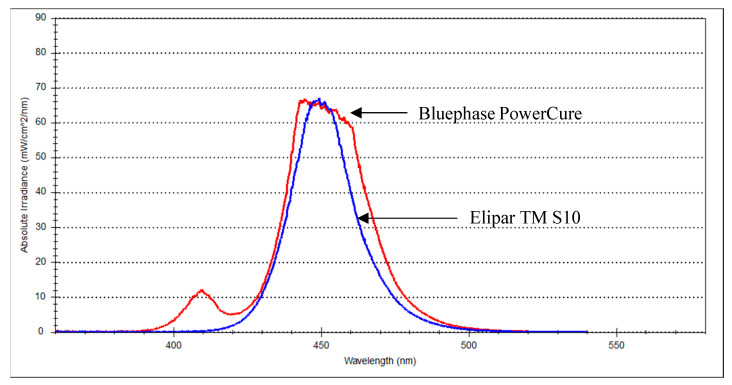
Spectral emission of both light curing units at 0 mm from the sensor.

As shown in Figure 1, the Elipar TM S10 emitted single-peak blue light, whereas the Bluephase PowerCure emitted two emission peaks of light, blue and violet. The difference (in percentage %) between each tested property of the material after being cured with either the recommended or the non-recommended light curing protocol is shown in the bar graph result of each test (Figure 1).

**Figure 2:**
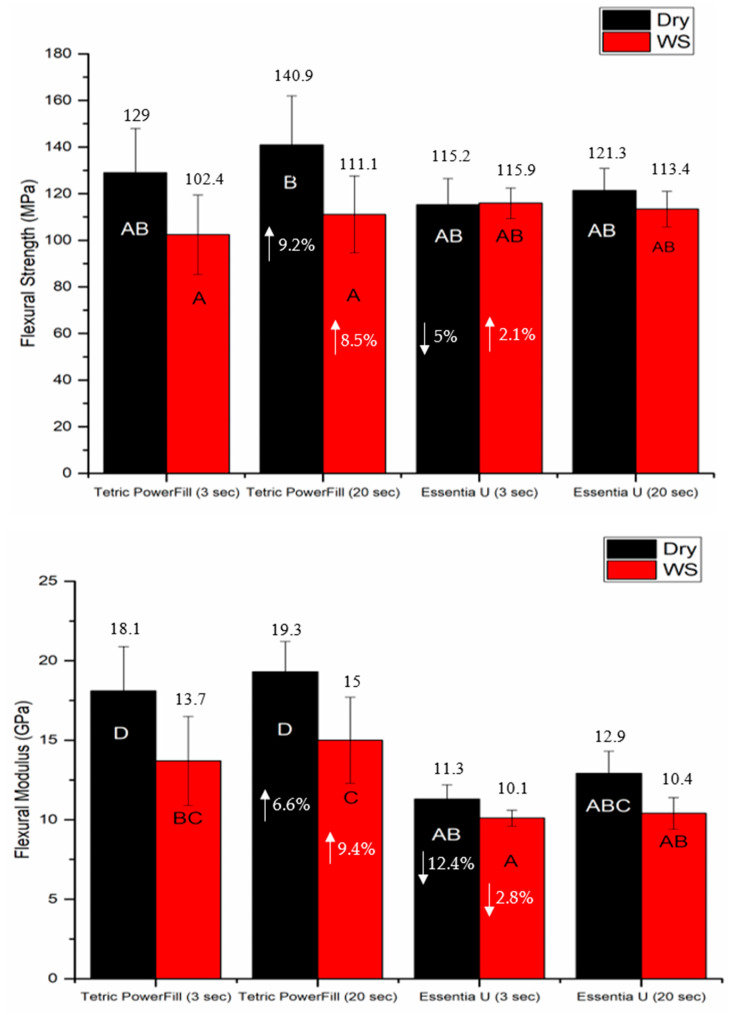
Bar graph showing means of flexural strength (MPa) and flexural modulus (GPa) with standard deviations (SD) of tested composite resins (WS refers to one month water storage at 37 °C). Non-statistically relevant variations (*p* > 0.05) between the materials are represented by the same letters within the bars. Differences between curing protocols presented as %.

**Figure 3:**
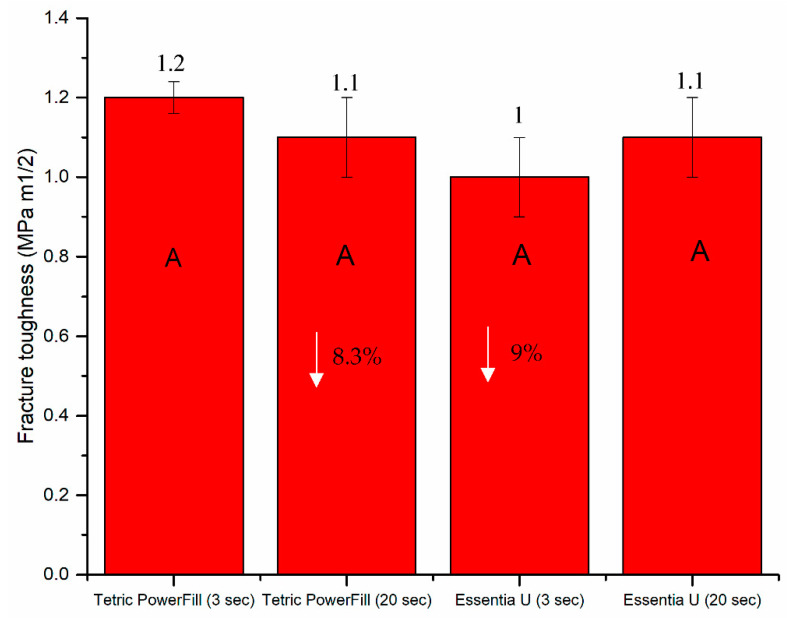
Bar graph showing mean fracture toughness (KIC) with standard deviations (SDs) of investigated composite resins. Non-statistically relevant variations (*p* > 0.05) between the materials are represented by the same letters within the bars. Differences between curing protocols presented as %.

The mean values of flexural strength, flexural modulus and fracture toughness for tested composite resins with standard deviations (SD) are summarized in Figure 2 and Figure 3. The data revealed that the curing protocol did not show a significant effect on the mechanical properties of both materials (*p* > 0.05).

**Figure 4:**
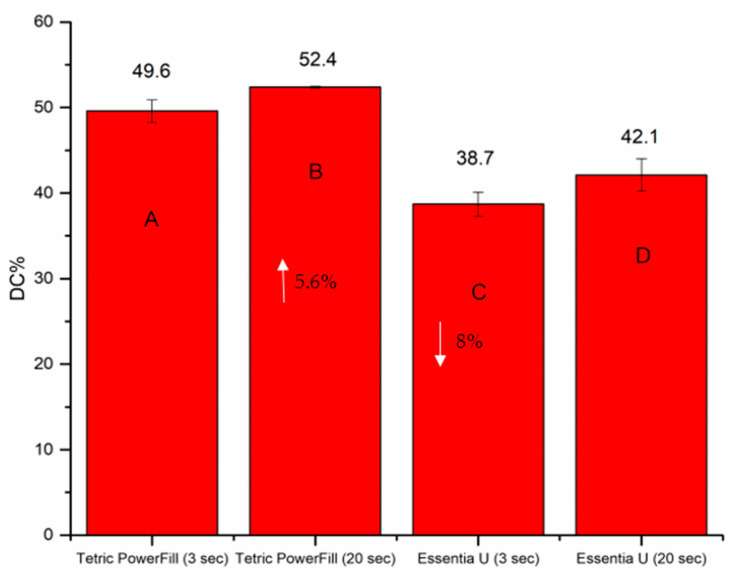
Bar graph illustrating means of the degree of conversion percentage (DC%) calculated at the bottom surface of the tested composite resins. Non-statistically relevant variations (*p* > 0.05) between the materials are represented by the same letters within the bars. Differences between curing protocols presented as %.

Water storage significantly decreased the flexural properties (*p* < 0.05) of the Tetric PowerFill group but not those of the Essentia U group (Figure 2). Despite the employed light curing protocol, Tetric PowerFill showed significantly (*p* < 0.05) higher flexural modulus and DC% than Essentia U. Tetric PowerFill with a conventional 20-s light curing protocol showed the highest DC% value (52.4) and Essentia U with a high-intensity (3-s) light curing protocol showed the lowest value (38.7) among all groups (*p* < 0.05) (Figure 4).

**Figure 5:**
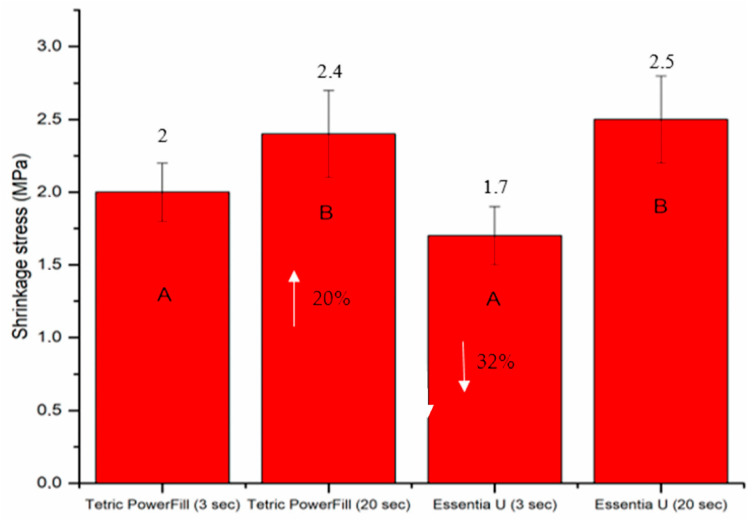
Bar graph showing means of the shrinkage stress (MPa) and standard deviations (SDs) of the investigated composite resins. Non-statistically relevant variations (*p* > 0.05) between the materials are represented by the same letters within the bars. Differences between curing protocols presented as %.

**Figure 6:**
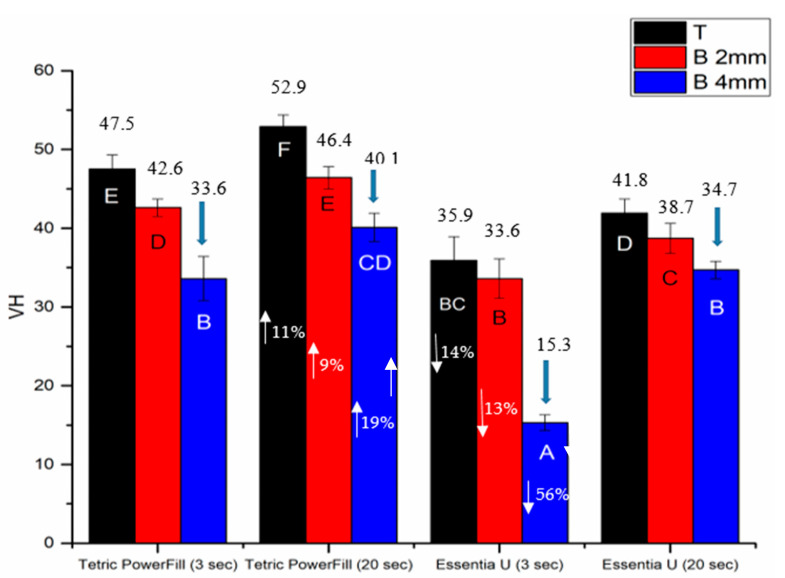
Bar graph showing means of surface microhardness (VH) at the top (T) and bottom (B) of 2- and 4-mm-thick specimens. Arrows above the columns indicate that the VH of these groups dropped below 80% of the top surface values. Non-statistically relevant variations (*p* > 0.05) between the materials are represented by the same letters within the bars. Differences between curing protocols at various thicknesses presented as %.

The DC%, shrinkage stress and surface microhardness (VH) of both composite resins significantly increased (*p* < 0.05) with the conventional 20-s light curing protocol (Figure 4, Figure 5 and Figure 6). Essentia U with both light curing protocols showed significantly lower VH at top and bottom surfaces than the Tetric PowerFill (*p* < 0.05). Concerning VH at the bottom of the 4-mm-thick specimens, all groups showed dramatic reductions in the VH values and the VH dropped below 80% of the top surface values (Figure 6).

**Figure 7:**
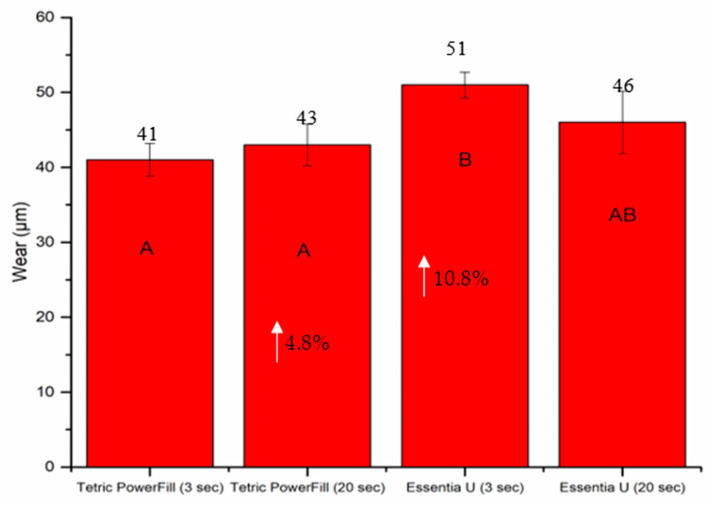

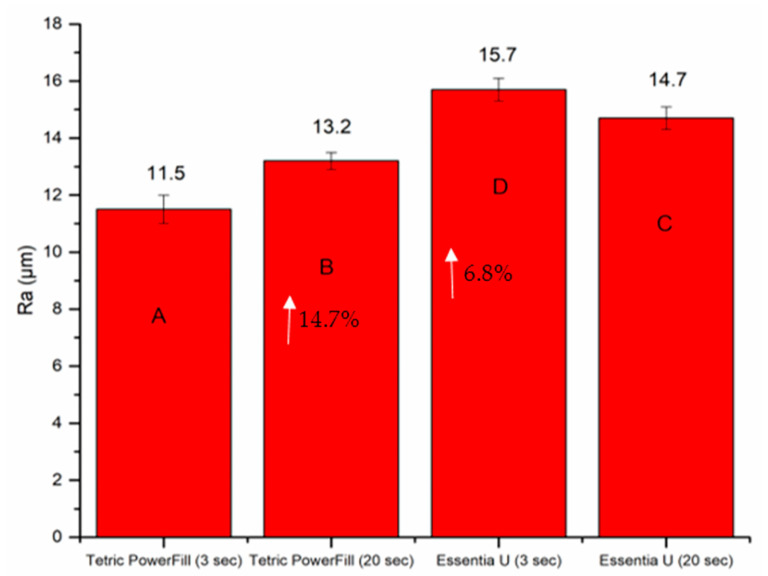
Bar graph showing means of wear depth (µm) and surface roughness (µm) with standard deviations (SDs) of investigated composite resins. Non-statistically relevant variations (*p* > 0.05) between the materials are represented by the same letters within the bars. Differences between curing protocols presented as %.

The mean values for wear depth and surface roughness (Ra) recorded for each composite resin after 15,000 chewing simulation cycles are shown in Figure 7. Regardless of the material and light curing protocol used, Ra after the wearing test was significantly higher (ranging between 11.5–15.7 µm) than before wearing (ranging between 0.2–0.4 µm) (*p* < 0.05). Essentia U with the 3-s light curing protocol presented the highest values (51 µm) of wear depth and Ra (15.7 µm) (*p* < 0.05) compared with all the tested groups (Figure 7).

**Figure 8:**
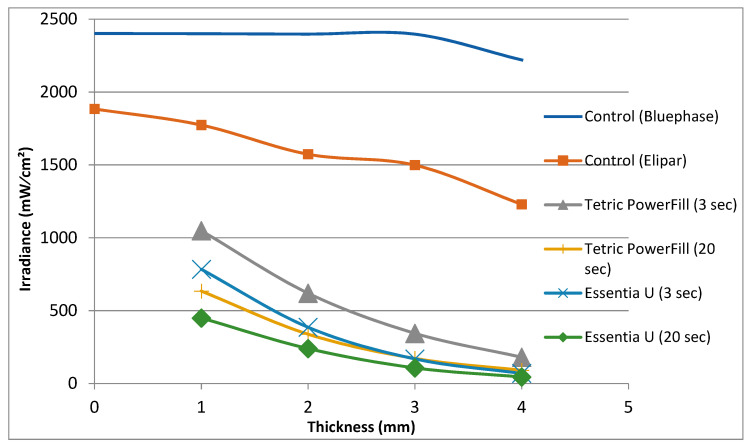
The irradiance (mW/cm^2^) of the light curing units at various thicknesses relative to the sensor through composite resins.

Both the light curing protocol and increment thickness of specimens had a significant effect on the irradiance of penetrating light values. As thickness increased, the irradiance value decreased for each group (Figure 8). The high-intensity (3-s) light curing protocol showed higher light transmission in both composite resins compared to the conventional light curing protocol (Figure 8).

**Figure 9:**
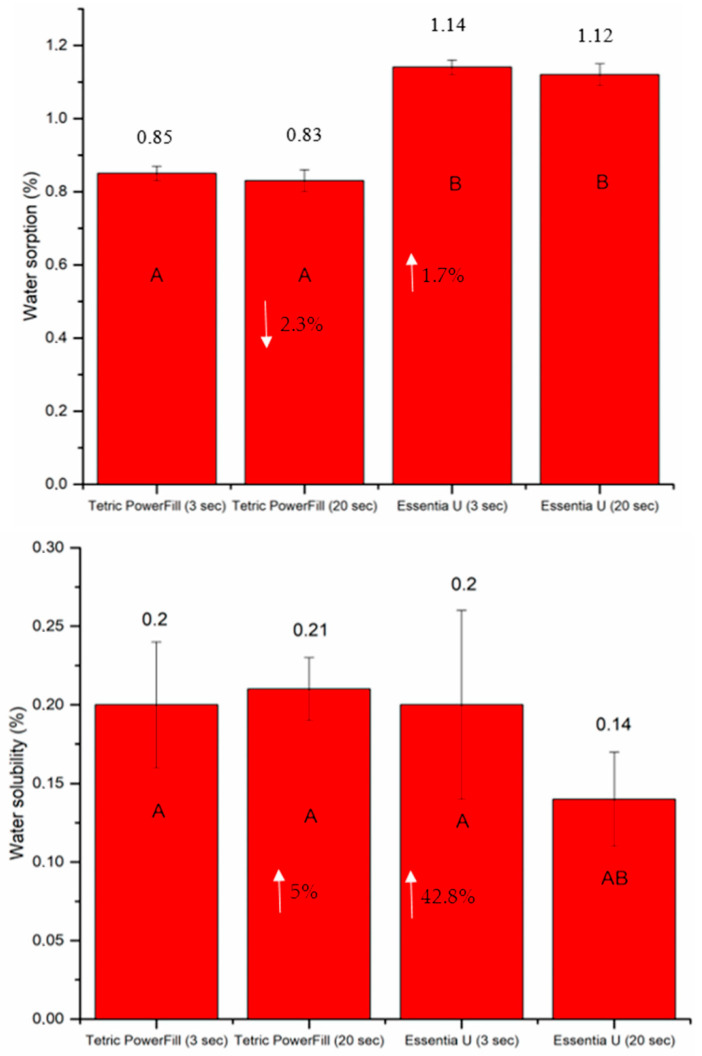
Bar graph showing means of water sorption and solubility with standard deviations (SDs) of tested composite resins during 30 days of storage in water at 37 °C. Non-statistically relevant variations (*p* > 0.05) between the materials are represented by the same letters within the bars. Differences between curing protocols presented as %.

As shown in Figure 9, Essentia U with both light curing protocols exhibited significantly (*p* < 0.05) higher water sorption values than Tetric PowerFill after 30 days of storage. On the other hand, the lowest values of water solubility were observed for Essentia U curing with the conventional 20-s light curing protocol (Figure 9).

**Figure 10:**
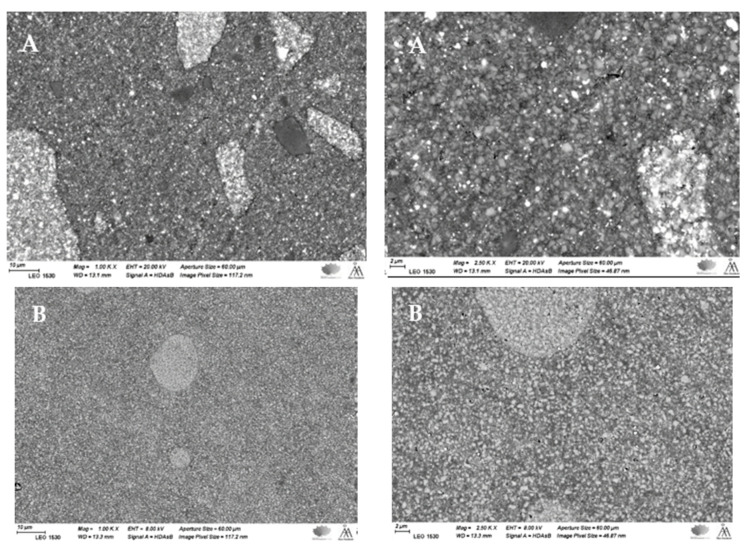
SEM photomicrographs (magnifications: 1000× and 2500×) of polished surface of investigated composite resins showing the filler sizes and distributions. (**A**) Tetric PowerFill and (**B**) Essentia U.

SEM examination revealed a typical microstructure of each tested composite with various particulate filler sizes and shapes in the composite resin matrix (Figure 10). This offered an additional justification for the dissimilar performance among the tested composite resins.

## 4. Discussion

In this investigation, most of the evaluated properties (physical, light transmission and wear) were impacted by the light curing protocol and material type. Therefore, the null hypotheses were rejected. The standard of restorative treatment may be severely undermined by using an unsuitable light curing protocol, without the practitioners recognizing their error until years later. This study was therefore designed to use two different composite resins from different manufacturers intended for different light curing protocols in order to reproduce the worst-case scenario of selecting the wrong material and curing protocol.

Because of this, the main goal of the current study was to give an overview of this critical clinical situation if a dental practitioner improperly used the PowerCure 3-s or conventional 20-s light curing protocol on the performance of composite resins. Thereby, our findings suggest that light curing a conventional (CQ-based) composite resin with a fast high-intensity (3-s) curing protocol results in some inferior material properties.

The composite resins must obtain adequate energy at the required wavelengths to achieve the optimal physical and surface properties [20]. Although there was a chemical difference between the two composite resins, both developed acceptable DC% (monitored for 15 min), confirming that when the curing protocol suggested by the manufacturers was used, adequate light was able to get to the bottom of the composite resin (Figure 4). On the other hand, CQ-based composite resin (Essentia U) light cured using the 3-s protocol showed reduced DC% values (38.7) due to decreased polymerization growth in the initial phases of the polymerization reaction. In other words, high intensity values can create extra radicals easily, and this can lead to more and earlier bi-radical polymer chain termination, resulting in regions where the DC% of the composite resin is lower [21]. This is in consistent with the work of Palin, et al. [22], who reported a low rate of polymerization and DC% of conventional CQ-based composite resin when cured using a fast high-intensity curing protocol. It is crucial to highlight that the rate of polymerization is affected not only by the energy dose, but also by the reactivity of the photoinitiator components, for instance the quantum yield, and additional causes [12].

In this study, Tetric PowerFill composite resin incorporated photointiators based on CQ, TPO and Ivocerin, in addition to AFCT reagent, in order to create a a more homogenous network. Ivocerin and diphenyl (2,4,6-trimethylbenzoyl) phosphine oxide (TPO) photoinitiators have been proven to be more reactive for high intensity light curing than the standard CQ/amine photoinitiator system [23,24]. In addition, by encouraging a more linear polymer chain growth, the AFCT compound reduces crosslinking density, moving the gel point to superior conversion values and eventually decreasing polymerization shrinkage stress [22]. This might be a justification for many results in this study including the DC% and shrinkage stress, and this is in line with previous research findings [10,25]. Therefore, it is not surprising that the composite resin (Essentia U), which exclusively contains the CQ/amine system, showed inferior values of DC%, VH, wear and surface roughness when polymerized with the 3-s light curing protocol.

The results of the tested mechanical properties showed that variations in material composition are a more significant source of variability than the curing protocol, and the obtained mechanical values were in agreement with previous studies [10,26,27].

On the basis of hardness measurements taken on the bottom and top surface of a light curing composite resin specimen, several studies in the literature have defined the curing depth, as depth of curing is closely related to the DC% values. Thereby, hardness values dropped with increasing depth and parts of the composite closer to the source of light sustain further DC% values and hence were harder [3,28]. The obtained hardness values were employed to measure a bottom/top hardness ratio, with a ratio of more than 80% being widely used as a minimum reasonable threshold value [3,29]. In view of this, both composite resins in this study could be used safely up to a 2-mm depth but not for a 4-mm depth (Figure 6). Because of the dramatic decrease in the VH values and the VH decline below 80% of the top surface values, this could be attributed to the minor quantity of light transmission across these composite resins (Figure 8). This result is in line with research by Par, et al. [30], and conflicts with the claim of the manufacturer that their bulk-fill composite resin (Tetric PowerFill) can be applied and cured in a 4-mm thick increment.

The quantity of light transmitted across a composite resin relies on the quantity of absorbed and scattered light [31]. This study displays that the light transmission across Tetric PowerFill (bulk-fill) with both light curing protocols was higher than for conventional composite resin (Essentia U) at all specimen thicknesses. The filler loading in Essentia U is higher and filler size is smaller (Figure 10) compared to those in Tetric PowerFill; this justifies the material’s decreased translucency. Previous research has shown that the light transmittance of composite resins can be affected by facts like the polymeric matrix refractive index, monomer form and filler type [32,33].

When composite resins are exposed to or stored in water, two separate processes occur. First, the water uptake leads to increased weight (sorption) and second, the leaching of ingredients occurs from the material in the mouth (solubility), resulting in a weight reduction [34]. Considering that Essentia U has smaller filler sizes, the current study’s water sorption results are consistent with those of Ilie and Hickel [35], who stated that the greater surface-area-to-volume ratio of the fillers in the nanofilled/nanohybrid materials appeared to raise the water uptake, even though the inclusion of nanofillers in the resin matrix enhanced the aesthetics to a significant degree. This was stated in relation to filler/matrix interface degradation [35]. On the other hand, the water solubility of Essentia U cured with the conventional 20-s light curing protocol was the lowest (Figure 9). This might be explained by the high crosslinking density of the composite matrix, which reduce the leaching of residual monomers [36].

The light curing protocols selected in this study were based upon the manufacturers’ instructions for the tested composite resins. Although the manufacturer’s irradiance value for the Bluephase PowerCure (3 s protocol) is 3050 mW/cm^2^, our measured value was around 2400 mW/cm^2^, which may be partially explained by the difference in measuring devices and the variance in diameter among the sensor and light curing tip. This is in harmony with the research by Shimokawa, et al. [37], who reported differences between the measured irradiance values of light curing units and those provided by the manufacturers. In addition to the irradiance value, it is essential to identify the emission spectrum of the light curing unit. In the present study, the Elipar TM S10 (1600 mW/cm^2^) emitted single-peak blue light, whereas the Bluephase PowerCure emitted two emission peaks of light, blue and violet (Figure 1).

Although the use of the PowerCure 3-s protocol during restorative procedures has been suggested to save working time, clinically, its light curing performance could be compromised by the difficulty of placing the light curing tip at a close distance to the composite resin being light cured, and the possibility of light curing tips that are scratched or polluted (with resin), apart from other substances, which would have a significant impact on the total quantity of power or energy supplied to the restorations. Moreover, properties such as surface gloss, color change, long-term water storage and biocompatibility should also be assessed in the future in order to demonstrate the whole picture of this tested clinical scenario.

## 5. Conclusions

We conclude that clinicians should use the high-intensity (3-s) or conventional (20-s) light curing protocol with only the composite resin specifically designed for this protocol in order to avoid undesirable clinical outcomes. The high-intensity (3-s) light curing protocol did not endanger the tested properties of Tetric PowerFill, except for the surface microhardness and water solubility. Although the light transmission through the bulk-fill composite resin (Tetric PowerFill) was higher than that of the conventional composite resin, the proper curing of composite resin in bulk increments requires further study.

## Figures and Tables

**Table 1:** The composite resins used in the study.

Material (Type & Shade)	Manufacturer	Composition
Tetric PowerFill (bulk-fill, IVA)	Ivoclar Vivadent AG, Liechtenstein (X48034)	Dimethacrylate monomers. Ba-Al-Silicate glass and Ytterbium trifluoride silica (inorganic fillers; Ø 0.04–3 μm with an average of 0.6 μm), 79 wt%
Essentia U (conventional, universal)	GC Corp, Tokyo, Japan (190122C)	UDMA, BisEMA, BisGMA, TEGDMA, Bis-MEPP. Prepolymerized silica (organic filler; Ø 16–17 µm) and barium glass (inorganic filler; Ø > 100 nm), 81 wt%
